# Functional Ag-EDTA-modified MnO_2_ nanocoral reef for rapid removal of hazardous copper from wastewater

**DOI:** 10.1007/s11356-023-30805-0

**Published:** 2023-11-22

**Authors:** Omnia I. Ali, Ahmed B. Azzam

**Affiliations:** Chemistry Department, Faculty of Science, Helwan University, Cairo, 11795 Egypt

**Keywords:** MnO_2_ nanostructures, EDTA-impregnation, Ag nanoparticles, Copper removal, Plackett–Burman design

## Abstract

A novel MnO_2_@EDTA-Ag nanocoral reef was constructed via a simplified redox reaction followed by EDTA and Ag nanoparticles impregnation to capture hazardous copper (II) from wastewater. A comprehensive characterization of the synthesized materials was conducted. The morphology of MnO_2_@EDTA-Ag in the form of a nanocoral reef was constructed of two-dimensional nanoplatelets and nanorod-like nanostructures. The optimal adsorption conditions proposed by the Plackett–Burman design (PBD) that would provide a removal % of 99.95 were pH 5.5, a contact time of 32.0 min, a Cu(II) concentration of 11.2 mg L^−1^, an adsorbent dose of 0.05 g, and a temperature of 40.3 °C. The loading of Ag nanoparticles onto MnO_2_@EDTA improved the adsorption capability of MnO_2_@EDTA-Ag. Additionally, the recyclability of MnO_2_@EDTA-Ag nanocoral reef was maintained at 80% after three adsorption–desorption cycles, and there was no significant change in the XRD analysis before and after the recycling process, implying its stability. It was found that nanocoral reef-assisted EDTA formed a chelation/complexation reaction between COO^−^ groups and C–N bonds of EDTA with Cu(II) ions. In addition, X-ray photoelectron spectroscopy (XPS) and Fourier transform infrared spectroscopy (FTIR) analysis proved the synergistic effect of the electrostatic interaction and chelation/complexation was responsible for the removal mechanism of Cu(II). Also, the results demonstrated no significant variation in MnO_2_@EDTA-Ag removal efficiency for all the tested real water samples, revealing its efficacy in wastewater treatment. Therefore, the current study suggests that MnO_2_@EDTA-Ag has substantial potential to be used as a feasible adsorbent for probable hazardous metals remediation.

**Supplementary Information:**

The online version contains supplementary material available at 10.1007/s11356-023-30805-0.

## Introduction

One of the most serious challenges facing the globe today is water scarcity, and resource depletion in some regions is leading to significant international conflict and human suffering (Asim et al. 2021; Lee et al. 2021). Wastewater treatment has been recognized as an essential concern over the past few decades, along with the growing industrialization of society (Fu et al. 2019; Elfiad et al. 2020). Heavy metal ions are frequently released by industrial waste, particularly in the metal coating, leather tanning, and petroleum refining industries. Clean water technologies have faced a major barrier for removing highly hazardous and ultra-dilute contaminants of concern. Due to its hazardous and non-biodegradable properties, copper is one of the most prevalent heavy metal contaminants in urban and agricultural water (Hama Aziz et al. 2023). Copper is derived from numerous industrial wastewaters, such as printed circuit boards (PCBs) and electroplating effluent (Chen and Xie 2020; Duan et al. 2023). The World Health Organization (WHO) has established a limit of 2 mg L^−1^ for copper ions in drinking water; however, the United States Environmental Protection Agency (USEPA) has set an allowed maximum of 1.3 mg L^−1^ for copper ions in industrial effluents (Al-Saydeh et al. 2017). A variety of physicochemical and biological methods, including electrodialysis, coagulation and flocculation, membrane filtration, and adsorption, have been used to decontaminate water that has been contaminated with potentially dangerous metals (Dastkhoon et al. 2018; Guo et al. 2021; Shi et al. 2021; Kuang et al. 2022). However, all approaches have deficiencies, such as the requirement for extensive amounts of chemicals and energy, limited separation selectivity, partial pollutant removal, and the production of sludge that must be safely disposed of (Markovski et al. 2014). Adsorption, one of the many methods for removing metal ions from water, is a quick and affordable process that makes it simple to regenerate and recover the separation medium (Hasanpour and Hatami 2020).

Owing to their enormous surface area, varied morphologies, and significant activity, nanosized metal oxides (NMOs) have a promising capacity to remove heavy metals from aquatic environments (Shi et al. 2021). Moreover, with their excellent adsorption capacity, selectivity, and oxidation abilities, nanostructured manganese oxides (MnO_2_) have been extensively employed for the removal of hazardous pollutants (Wei et al. 2019; Husnain et al. 2020). One of the most diverse groups of porous materials, MnO_2_ includes nanorods, nanowires, nanoflowers, nanotubes, nanoparticles, and nanosheets among its many different structures and morphologies (Mallakpour and Motirasoul 2017). It has numerous crystallographic forms, such as α, β, γ, δ, ε, and λ-type, that are different in the spatial arrangement of MnO_6_ octahedral units (Ghosh 2020). Due to their exceptional structural flexibility and new physical and chemical properties, manganese dioxides and their derivative compounds have received a lot of attention. Therefore, they can be utilized in many scientific and industrial applications, such as fuel cells, energy storage devices (supercapacitors and rechargeable batteries), catalysis, sensors, and adsorbents (Zhang et al. 2015; Zhao et al. 2017; Liu et al. 2018; Husnain et al. 2020; Bigiani et al. 2020; Hao et al. 2020; Asim et al. 2021; Claros et al. 2021). However, MnO_2_ nanostructures have a significant tendency to adhesion and aggregation because of their high energy surface, small size, and electrostatic forces that limit their performance. Surface enhancements that are chemical and physical can solve this problem (Mallakpour and Motirasoul 2017). Therefore, we employed EDTA to modify the surface of MnO_2_ with the coordination of carboxylic groups (–COOH) to the surface of MnO_2_. EDTA is a metal-chelating agent with high intrinsic selectivity that is commonly attached to substrate surfaces for application. Chen et al. prepared Fe_3_O_4_-MnO_2_-EDTA magnetic nanoparticles for targeted Cu(II) adsorption in a complex system (Chen and Xie 2020). EDTA-modified composites have comparable separation challenges as traditional adsorbents. However, common chelating agent EDTA has the ability to form stable chelates with metal ions and has good degradability in certain environmental systems, which can prevent secondary pollution (Repo et al. 2010; Panahandeh et al. 2021). Noble metals have recently received a lot of attention for their outstanding adsorption abilities. Due to their unique qualities, such as closely packed structure, varied crystallographic facets, and high surface-to-volume ratio, Ag nanoparticles (NPs) have been widely used as a possible adsorbent for industrial applications (Song et al. 2016; Vicente-Martínez et al. 2020). However, its reusability in large-scale wastewater treatment applications is constrained by NPs aggregation and challenging recovery. Therefore, employing effective assistance for Ag NPs may help in resolving these issues. An interesting candidate for use as an adsorbent material is the direct incorporation of Ag NPs on other active materials such as MnO_2_. However, the direct immobilization of Ag NPs onto active materials, such as MnO_2_, causes repulsive forces that need to be overcome to address stability. The issue of repulsive forces can be solved by incorporation an organic buffer layer between the two active materials, which will also provide a strong synergy in the hybrid structure, allowing it to be employed as an efficient adsorbent (Sharif et al. 2019).

Herein, a novel MnO_2_@EDTA-Ag nanocoral reef was effectively synthesized via a facile redox reaction followed by impregnation with EDTA and silver nanoparticles for rapid removal of hazardous copper (II) from real water samples. The dissolution of the MnO_2_ adsorbent was avoided by impregnation with EDTA and Ag nanoparticles. Therefore, the feasibility of our strategy provides essential support for stability evaluation towards Cu(II) ions with acceptable adsorption properties. First, coral reef-like MnO_2_ nanoparticles were prepared via a rapid redox reaction between KMnO_4_ and Mn(II) sulfate. The dissolution of MnO_2_ adsorbent was avoided by impregnation with EDTA and Ag nanoparticles as well. Statistical methods of experimental design, including factorial design and response surface analysis, have been employed in different methods for heavy metals’ removal because of their ability to reduce the limitations of the time-consuming single-factor conventional and classical methods. For example, the Plackett–Burman design is a quick and useful screening technique to find the significant parameters amid a large number of variables, ensuring that each parameter is well understood, and saving time as well (Taylor et al. 2012; El-naggar et al. 2018). Therefore, the influences of all studied parameters on the removal efficiency of Cu(II) using MnO_2_@EDTA-Ag were investigated systematically using the Plackett–Burman design. A statistical analysis of the obtained results was carried out using ANOVA to evaluate the inference and impact of the studied parameters on copper removal. In addition, the impact of operating parameters, such as the initial pH, contact time, dose of adsorbent, initial metal concentration, temperature, and interfering ions towards Cu(II) removal was explored by adopting batch mode. To further assess the sorption behavior of MnO_2_@EDTA-Ag, the kinetics, rate-controlling mechanisms, isotherms, and thermodynamic characteristics of the sorption process have also been examined. Additionally, the desorption study, stability evaluation, and feasible mechanistic pathway for Cu(II) removal were elucidated.

## Experimental

All materials and the characterization methodologies are provided in the electronic supplementary information (ESI).

### Synthesis of MnO_2_ nanostructures

A simple, ultrafast, economical, and environmentally friendly redox reaction was developed for the construction of nanocoral reef-like MnO_2_. In a 500-mL beaker, 4.61 g of MnSO_4_ was combined with 100 mL of isopropyl alcohol (IPA). The solution was vigorously stirred while being heated to around 60 °C. After being thoroughly dissolved in 150 mL of double-distilled water, 3.00 g of KMnO_4_ was quickly added to the previously mentioned boiling solution while being stirred for 10 min. Immediately, a significant amount of brown-black precipitate was collected. After being centrifuged, the mixture was cleansed with double-distilled water and oven-dried for 6 h at 90 °C. Under the same conditions, various solvents were used to create additional MnO_2_ structures. Typical solvents included glycerol and ethylene glycol.

### Synthesis of Ag-EDTA-modified MnO_2_ nanostructures

The above-obtained brown-black precipitate MnO_2_ (100 mg) prepared by isopropyl alcohol (IPA) as a solvent was dispersed in 20 mL H_2_O and followed by 100 mg of EDTA. The mixture was mixed for 2 h and then agitated for 48 h at room temperature. The resulting EDTA-modified MnO_2_ nanostructures were labelled as MnO_2_@EDTA. A simple sonochemical method was developed to construct embedded Ag nanoparticles on MnO_2_@EDTA. In order to create the Ag–NH_4_^+^ solution, 150 mg of AgNO_3_ was combined with 25 mL of NH_4_OH (5%) in 150 mL of water. Afterward, 150 mL of Ag–NH_4_^+^ solution was sonicated with 50 mg of MnO_2_@EDTA for 10 min at room temperature. Finally, the grayish-black solid product (MnO_2_@EDTA-Ag) was centrifugated, washed with dilute HNO_3_, and double-distilled water several times, then dried at a temperature of 70 °C.

### Statistical experimental design

For studying Cu(II) removal using MnO_2_@EDTA-Ag, the removal percentage of metal could depend on the medium pH, the initial Cu(II) concentration (*C*_o_), the contact time (*t*), the adsorbent dose (*m*), and the temperature. Plackett–Burman design was employed to determine the significant parameters for Cu(II) removal. The low and high levels are given in Table 1. Design expert statistical software program, version 12, was employed for the experimental design and data statistical analysis.

**Table 1:** Levels of the parameters tested in Plackett–Burman design

Parameter	Symbol	Level	
		Low (− 1)	High (+ 1)
pH	A	2	6
Time (min)	B	1	60
Concentration of Cu(II) (mg L^−1^)	C	10	50
Adsorbent dose (g)	D	0.001	0.1
Temperature (°C)	F	25	55

### Adsorption experiments

Batch adsorption experiments were conducted at a constant rate of 150 rpm in a thermostatic shaking water bath (AHAAM, Egypt). The impacts of several parameters were studied, such as initial pH (2–6), contact time (1–120 min), initial Cu(II) concentration (10–50 mg L^−1^), the adsorbent dose (0.001–0.1 g), and temperature (25–55 °C) using 10 mL of Cu(II) solutions. The pH values of the copper solutions were altered as required through the addition of drops of HCl (0.1 mol L^−1^) or NaOH (0.1 mol L^−1^). The values of the point of zero charges (pH_PZC_) of the prepared adsorbents were calculated using the salt addition method (Bakatula et al. 2018). After a measured time interval, samples were taken out and filtered through a membrane syringe filter (0.22 μm) to be analyzed to determine the residual Cu(II) concentration using the modified sodium diethyldithiocarbamate method (Ali and Mohamed 2017).

The removal efficiency (*R*%) and the amount of Cu(II) adsorbed (*q*_e_, mg g^−1^) were calculated using the following equations. (Mozaffari et al. 2020b, c):

1 $$R\mathbf{\%}=\frac{\left({C}_{\mathrm{o}}- {C}_{\mathrm{e}}\right)}{{C}_{\mathrm{o}}}\times 100$$

2 $$q_{\mathrm e}\left(\mathrm{mg}\;\mathrm g^{-1}\right)=\frac{\left(C_{\mathrm o}-C_{\mathrm e}\right)\times V}m$$

where *C*_o_ and *C*_e_ (mg L^−1^) represent the initial and equilibrium copper concentrations, respectively; *V* is the volume (*L*) of the copper solution, and *m* denotes the mass (g) of the adsorbent.

### Desorption and reusability

After adsorption of 10 mL Cu(II) (10 mg L^−1^) using 0.01 g of MnO_2_@EDTA-Ag, the loaded adsorbent was filtered and dried. The adsorbed Cu(II) was desorbed using different concentrations (0.001, 0.005, and 0.01 mol L^−1^) of different acids (HCl, HNO_3_, and H_2_SO_4_). The desorbed Cu(II) was separated and analyzed as described in the “Adsorption experiments” section. Desorption efficiency (*E*_Des_) was estimated using Eq. (3) (Ali et al. 2020):

3 $${E}_{\mathrm{Des}}=\frac{{D}_{\mathrm{Des}}}{{A}_{\mathrm{Ads}}}\times 100$$

where the amounts of adsorbed and desorbed Cu(II) are represented by *A*_Ads_ and *D*_Des_, respectively. The regenerated adsorbent was employed in the following adsorption tests for reusability, and the adsorption–desorption cycle was repeated with the same sample. The produced adsorbent’s ability to capture Cu(II) ions from a variety of real water samples was next tested analytically. Several water samples were used, including tap water, groundwater, and Nile water.

## Results and discussion

### Characterization

**Fig. 1:**
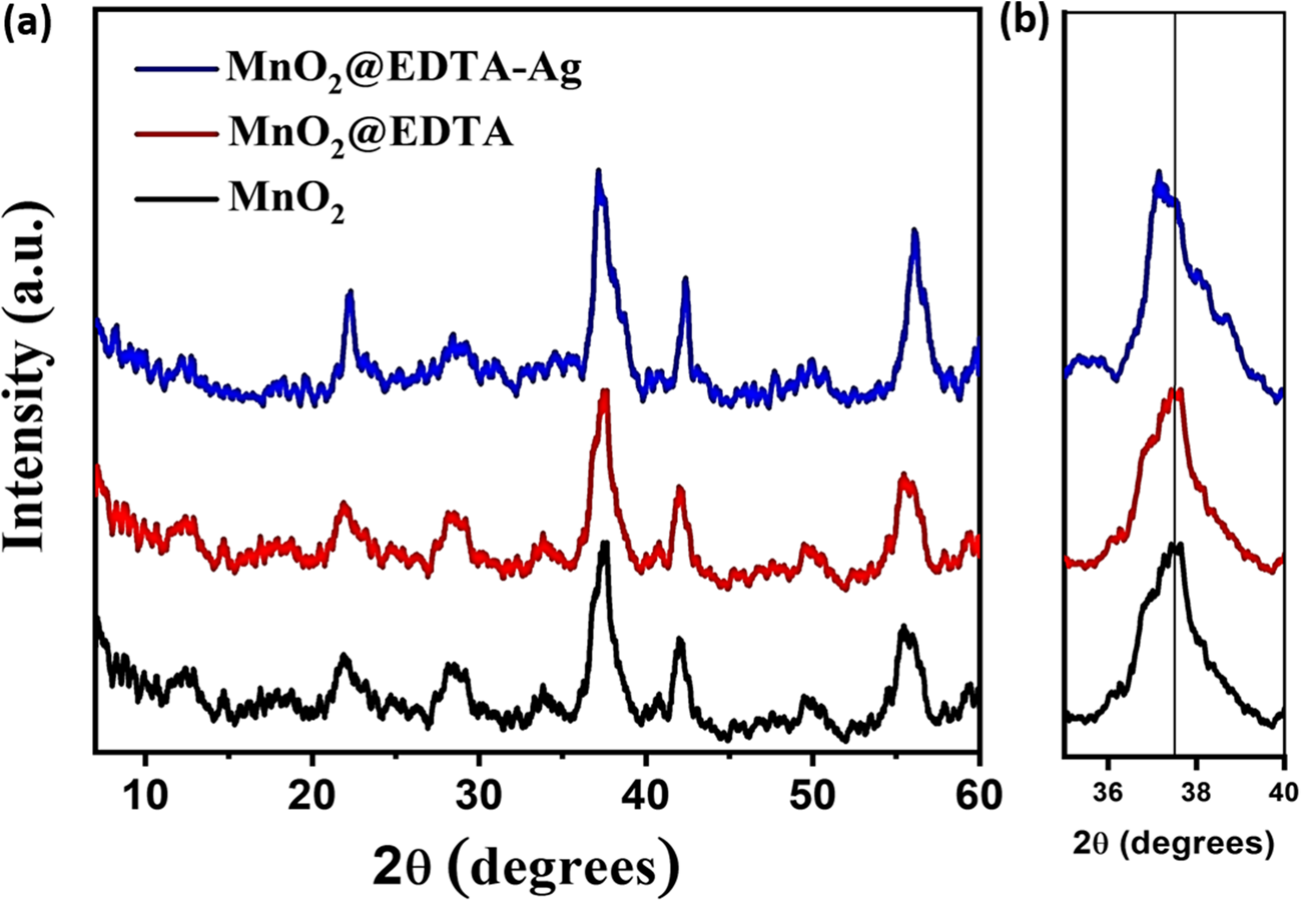
XRD patterns of MnO_2_, MnO_2_@EDTA, and MnO_2_@EDTA-Ag nanocoral reef

The XRD patterns of the MnO_2_, MnO_2_@EDTA, and MnO_2_@EDTA-Ag samples are shown in Fig. 1 a. The figure shows that all of the samples had heterogeneous crystalline forms of manganese oxide: a major phase of γ-MnO_2_ with a minor phase of β-MnO_2_. The diffraction peaks at about 21.87°, 37.43°, 41.97°, and 55.56° corresponded to (120), (131), (300), and (160) lattice planes of γ-MnO_2_ (JCPDS 14–0644). On the other hand, the diffraction peak at about 28.5° for the MnO_2_ sample corresponded to the reflection plane of (110) of a pyrolusite-type (β-MnO_2_) of manganese oxide (JCPDS 24–0735) (Devaraj and Munichandraiah 2008; Bai et al. 2016; Raheem and Al Sammarraie 2020; Sun et al. 2020). It can be observed that there is no significant difference in the XRD pattern of MnO_2_@EDTA upon the modification of the MnO_2_ sample with EDTA, demonstrating that the phase structure was almost completely conserved. However, with the incorporation of Ag nanoparticles, it can be seen that the peaks became sharper and more intense (Fig. 1b), reflecting the higher crystallinity of MnO_2_@EDTA-Ag sample. In addition, the XRD pattern of MnO_2_@EDTA-Ag lacked the expected Ag diffraction peaks, which may be attributable to the great dispersion of Ag nanoparticles.

**Fig. 2:**
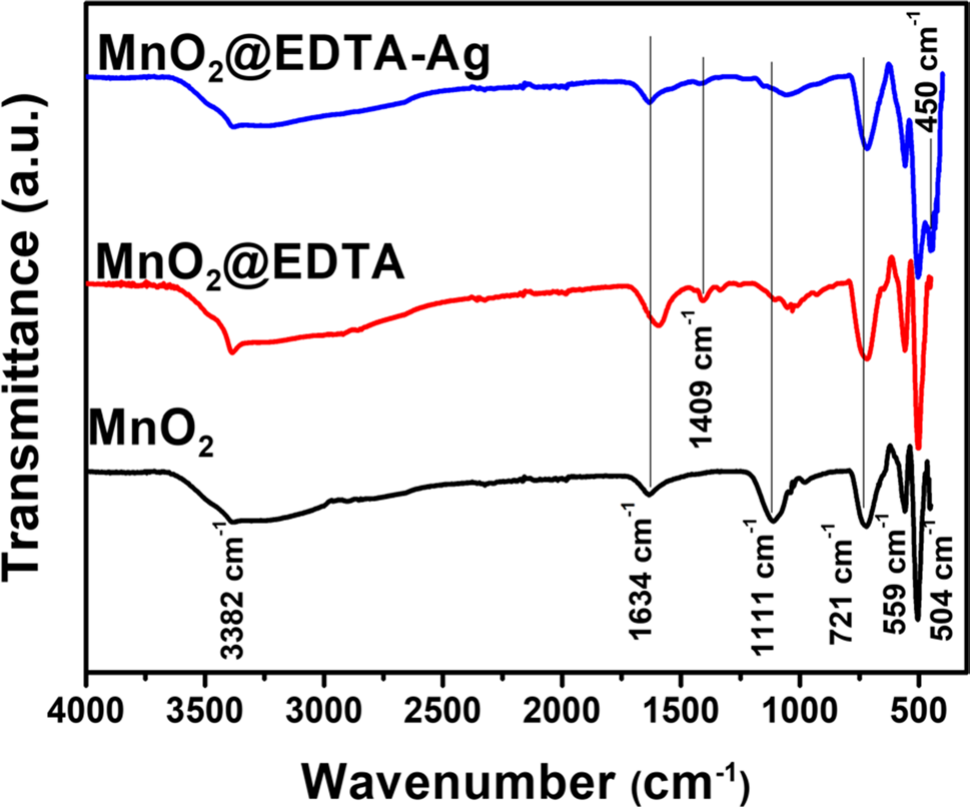
FTIR spectra of MnO_2_, MnO_2_@EDTA, and MnO_2_@EDTA-Ag nanocoral reef

The surface functional groups of MnO_2_, MnO_2_@EDTA, and MnO_2_@EDTA-Ag nanocoral reef were verified by FT-IR spectra as shown in Fig. 2. The peaks located around 500 and 720 cm^−1^ are associated with the Mn–O vibration of [MnO_6_] octahedra (Asim et al. 2021). A new peak at 1409 cm^−1^ is observed in MnO_2_@EDTA and MnO_2_@EDTA-Ag samples, and it is attributed to the symmetric stretching vibration of C–N bonds and COO^−^ groups derived from EDTA (Chen and Xie 2020). This new peak is absent in the MnO_2_ sample, indicating that EDTA was successfully anchored on MnO_2_@EDTA. The non-existence of this peak in MnO_2_ sample is evidence of the successful anchoring of EDTA in the MnO_2_@EDTA sample. In addition, a small peak that appeared at 450 cm^−1^ is assigned to Ag, confirming that Ag nanoparticles have been successfully embedded into MnO_2_@EDTA (Sharif et al. 2019). The bending vibrations of the –OH groups associated with the Mn atoms appeared at a peak of 1634 cm^−1^ (Dinh et al. 2020). The broad vibration band around 3382 cm^−1^ represents O–H stretching vibration, which is from water molecules that are physically adsorbed to the surface of samples (Revathi and Kumar 2017; Cao et al. 2021).

**Fig. 3:**
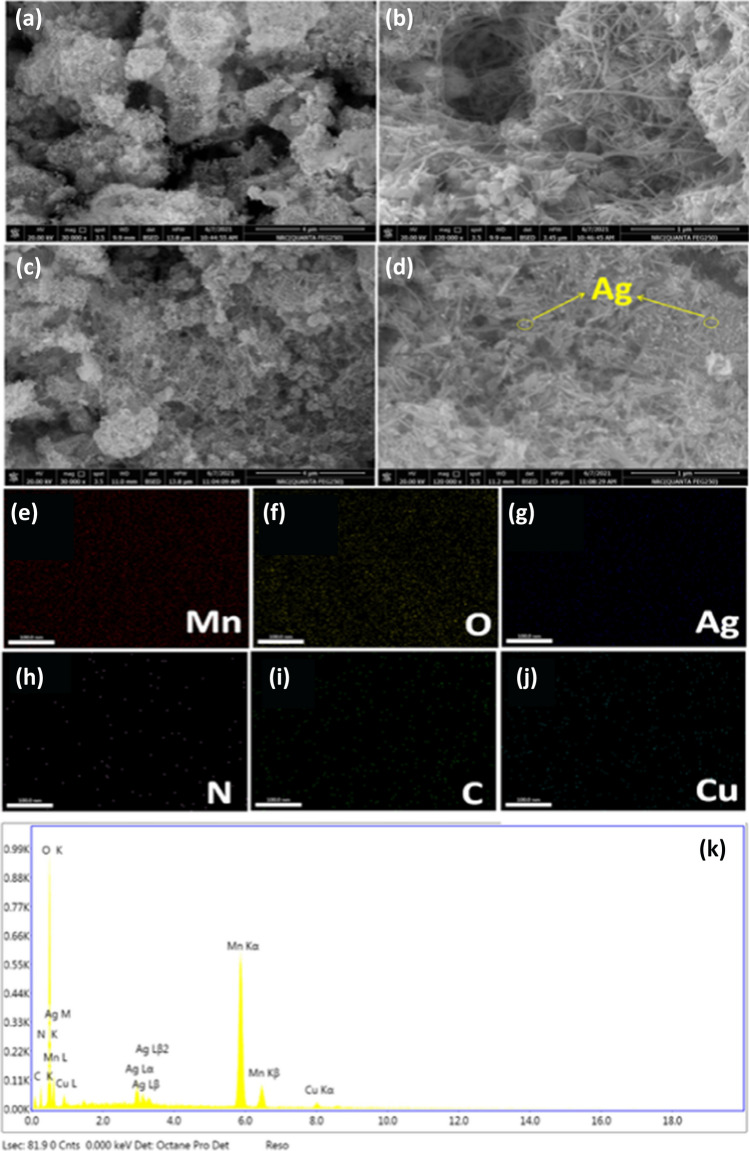
**a**–**b** FESEM images of MnO_2_@EDTA nanocoral reef; **c**–**d** MnO_2_@EDTA-Ag nanocoral reef; **e**–**j** elemental mapping of MnO_2_@EDTA-Ag nanocoral reef after copper removal showing Mn, O, Ag, N, C, and Cu; **k** EDX of MnO_2_@EDTA-Ag nanocoral reef

**Fig. 4:**
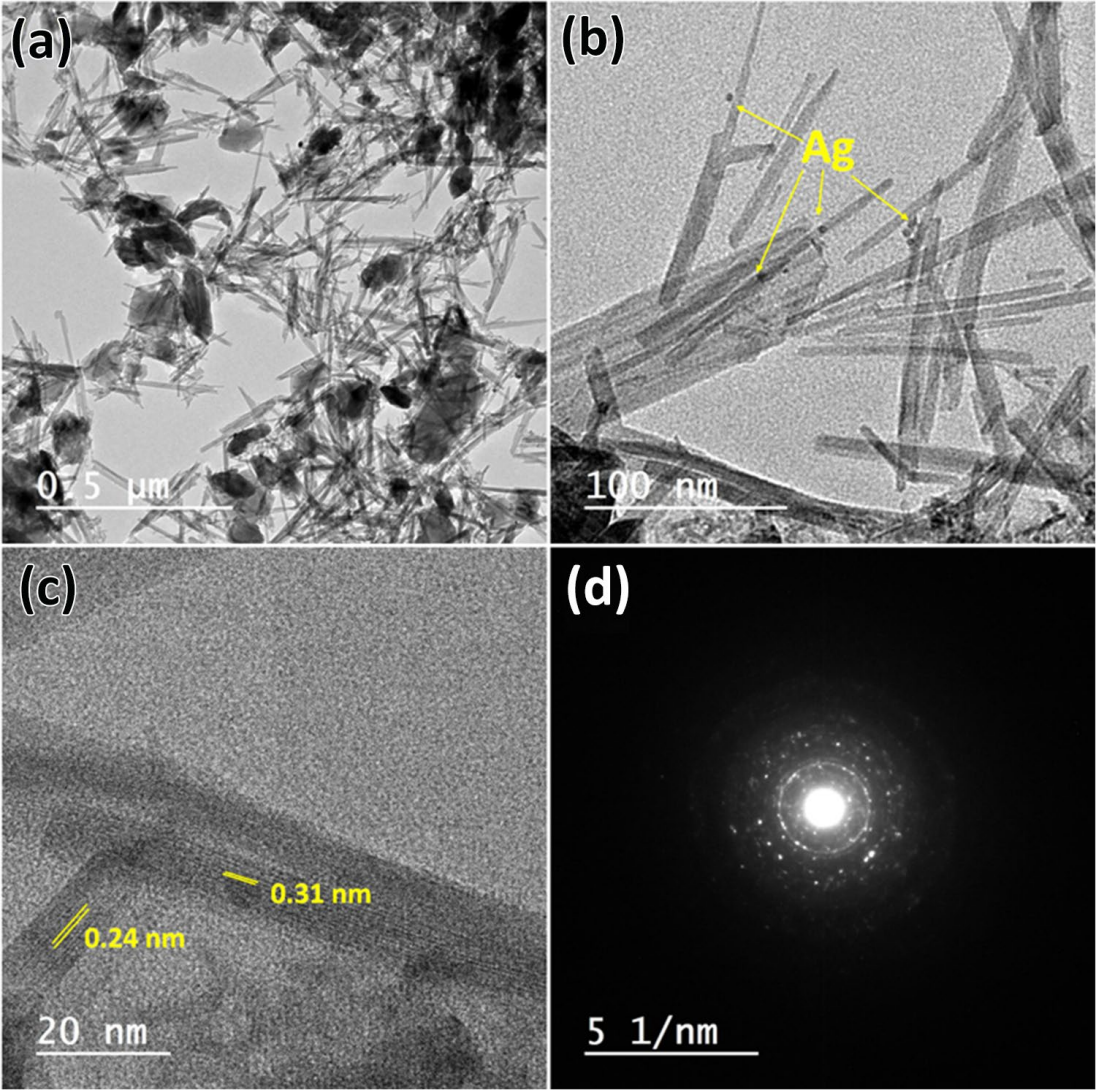
**a**–**b** TEM images, MnO_2_@EDTA-Ag; **c** HRTEM; **d** SEAD pattern

The surface morphologies of MnO_2_@EDTA and MnO_2_@EDTA-Ag structures were characterized by FESEM. As displayed in Fig. 3a–b, MnO_2_@EDTA shows a nanocoral reef composed of a two-dimensional nanoplatelet structure with the formation of some nanorods out of the plate structure. Figure 3c–d clearly show the in situ deposition of Ag (NPs) on the surface of the MnO_2_@EDTA nanocoral reef. The elemental mapping analysis (Fig. 3e–j) indicated that Mn, O, C, N, and Ag elements were homogeneously distributed in the hybrid structure. The EDX spectrum of MnO_2_@EDTA-Ag is shown in Fig. 3g, and the elemental composition confirms the existence of Mn, O, C, N, and Ag elements. In the meantime, the TEM images (Fig. 4a–b) also confirmed that the obtained MnO_2_@EDTA-Ag was a nanocoral reef composed of two-dimensional nanoplatelet with nanorod-like structure, which was the same as the SEM results. The average diameter of a nanoplatelet is 60 nm, and the diameters of nanorods are about 10 to 80 nm and 200 nm in length. In addition, the embedded Ag NPs were observed and attached to the surface nanorods (Fig. 4b). HRTEM image (Fig. 4c) of MnO_2_@EDTA-Ag nanocoral reef exhibited lattice fringes of 0.24 and 0.31 nm. These spacings are related to the (131) lattice plane of the γ-MnO_2_ phase and the (110) lattice plane of the β-MnO_2_ nanostructures, as shown in Fig. 4c. It can be concluded that the Ag NPs were successfully immobilized on the MnO_2_@EDTA nanocoral reef to form a hybrid structure. Figure 4 d illustrates the SAED pattern of the MnO_2_@EDTA-Ag nanocoral reef, which demonstrates the polycrystalline nature of the hybrid structure.

### Statistical experimental design

Plackett–Burman design (PBD) using two-level factors was utilized to analyze the removal of Cu(II) ions employing MnO_2_@EDTA-Ag taking into consideration different parameters: pH (A), contact time (B), initial Cu(II) concentration (C), adsorbent dose (D), and temperature (E). Table 2 summarizes the different operating parameters along with the experimental results of *R*% for Cu(II) removal using MnO_2_@EDTA-Ag according to PBD experiments. The data listed in the table indicated a substantial variation in Cu(II) removal efficiency. The lowest *R*% of 8.34% was achieved using a MnO_2_@EDTA-Ag dose of 0.005 g, 1 min as contact time, 50 mg L^−1^ Cu(II), pH 3, and at 55 °C. In contrast, the highest *R*% (95%) was reached with a MnO_2_@EDTA-Ag dose of 0.05 g, 90 min as a contact time, 10 mg L^−1^ Cu(II), pH 6, and at 55 °C. The residuals between the practical (actual) and predicted (model) values were found to be small, indicating that both the observed and predicted responses had satisfactory agreement.

**Table 2:** Results of Cu(II) removal using MnO_2_@EDTA-Ag using Plackett–Burman design

Run no	pH	Time (min)	Conc. (mg L^−1^)	Adsorbent dose (g)	Temp. (°C)	Experimental *R*%	Predicted *R*%	Residuals
1	6	90	10	0.005	25	51.59	51.68	− 0.138
2	3	90	50	0.005	55	15.8	16.86	− 1.039
3	6	1	50	0.05	25	73.34	74.40	− 1.039
4	3	1	10	0.005	25	42.99	43.29	− 0.214
5	3	90	10	0.05	55	85	83.38	1.586
6	6	90	50	0.005	25	33	31.38	1.586
7	3	1	10	0.05	25	83.12	82.73	0.378
8	6	90	10	0.05	55	95	96.54	− 1.605
9	3	1	50	0.005	55	8.34	8.43	− 0.138
10	3	90	50	0.05	25	57.1	57.66	− 0.447
11	6	1	50	0.05	55	74.2	73.05	1.201
12	6	1	10	0.005	55	73.8	73.89	− 0.138

**Fig. 5:**
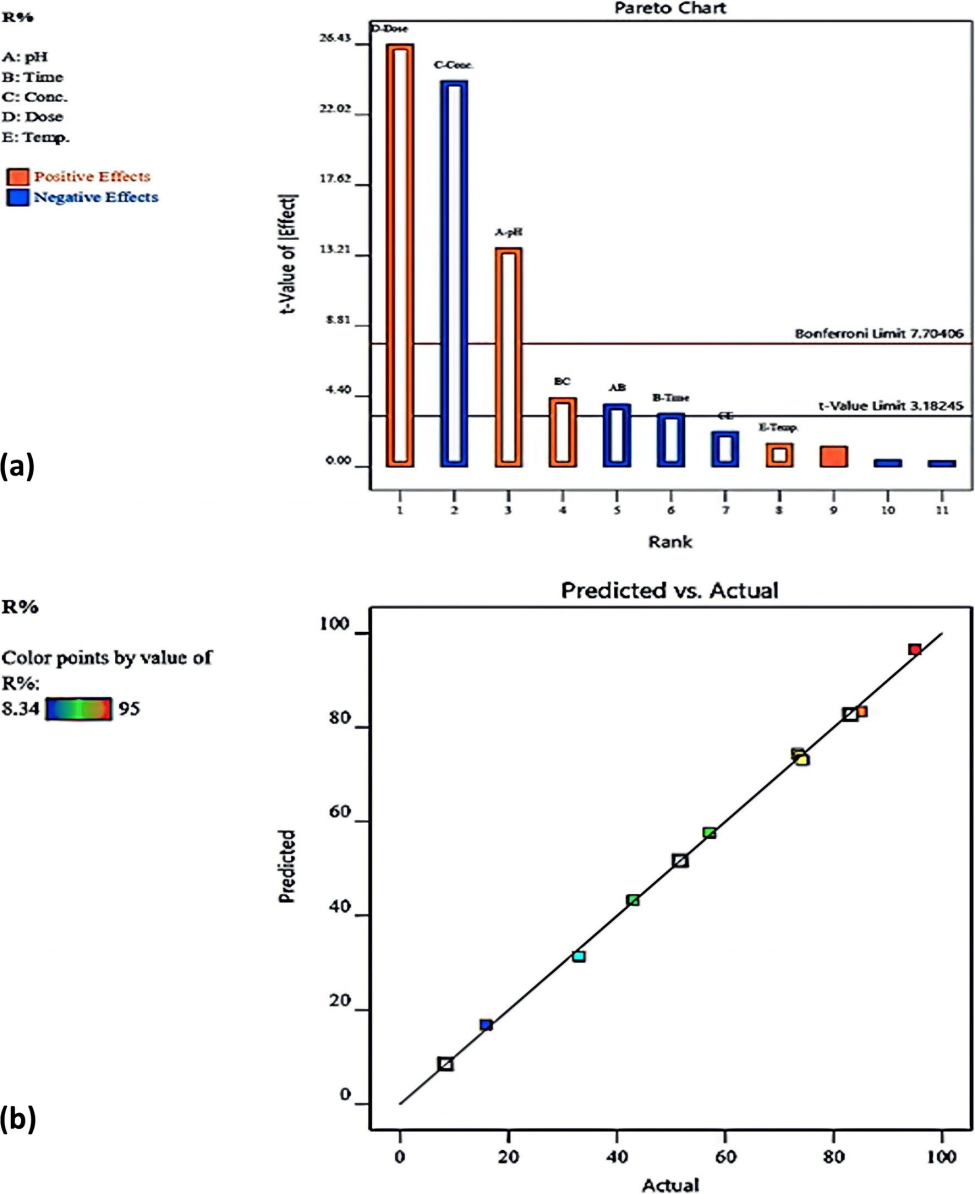
**a** Pareto chart of the impacts of various parameters on the Cu(II) removal and **b** graphical representation of experimental values as a function of predicted values

Analysis of variance (ANOVA) was employed to assess the appropriateness of the model. The ANOVA of R% of Cu(II) using MnO_2_@EDTA-Ag is exhibited in Table 3. The model is highly significant in this study, as shown by the *F* value of 273.61, and there was only a 0.03% possibility that the results were only due to noise. The correlation coefficient (*R*^2^) manipulated to reveal the relationship between the experimental (actual) and predicted responses was 0.9986, as confirmed by the model. This implies that the process parameters analyzed justify 99.86% of the removal efficiency variability. Moreover, as displayed in the table, the value of the predicted *R*^2^ (0.9791) is in acceptable agreement with the adjusted *R*^2^ (0.9950), i.e., the deviation is < 0.2. Also, model terms with *P*-values < 0.05 imply that they are statistically significant. The dose (D) of MnO_2_@EDTA-Ag was the most significant parameter affecting Cu(II) removal, followed by Cu(II) concentration (C) > pH (A) >> time (B) and their interactions (AB and BC). On the other hand, temperature (E) and interaction (CE) had no significant impact on Cu(II) removal. As demonstrated in Table 3, the respective contributions of the investigated parameters, including pH, time, Cu(II) concentration, adsorbent dose, and temperature, were 31.7, 30.51, 25.17, 12.44, and 0.18%, respectively. In addition, a Pareto chart was employed to affirm the impact of each parameter on Cu(II) removal (Fig. 5a). As demonstrated, pH and adsorbent dose had positive effects on the Cu(II) removal efficiency, whereas time and Cu(II) concentration had negative effects. The following equation can be utilized to figure out the model equation of R% for copper removal:

**Table 3:** Analysis of variance for the experimental results of the PBD

Source	Sum of squares	df*	Mean squares	*F*-value	*p*-value	Contribution %
%Model	8603.16	8	1075.39	273.61	0.0003	
A-pH	734.98	1	734.98	187.00	0.0008	12.27
B-Time	43.65	1	43.65	11.10	0.0446	0.73
C-Conc	2293.71	1	2293.71	583.58	0.0002	38.29
D-Dose	2745.49	1	2745.49	698.52	0.0001	45.83
E-Temp	8.26	1	8.26	2.10	0.2431	0.14
AB	60.20	1	60.20	15.32	0.0297	
BC	72.71	1	72.71	18.50	0.0231	
CE	19.10	1	19.10	4.86	0.1147	

^*^df: degrees of freedom, *R*^2^ = 0.9986, adjusted *R*^2^ = 0.9950, predicted *R*^2^ = 0.9791

4 $$R\mathrm{\% }= 57.77 + 9.59 \times \mathrm{ pH }- 2.09 \times \mathrm{ Time }- 15.15 \times \mathrm{ Conc}. + 19.72 \times \mathrm{ Dose }+ 1.02 \times \mathrm{ Temp}. - 3.00 \times \mathrm{ pH }\times \mathrm{ Time }+ 3.30 \times \mathrm{ Time }\times \mathrm{ Conc}. - 1.69 \times \mathrm{ Conc}. \times \mathrm{ Temp}.$$

Figure 5b displays the plot between the actual values and predicted values of the removal efficiency of Cu(II). As demonstrated, both values are in reasonable agreement.

**Fig. 6:**
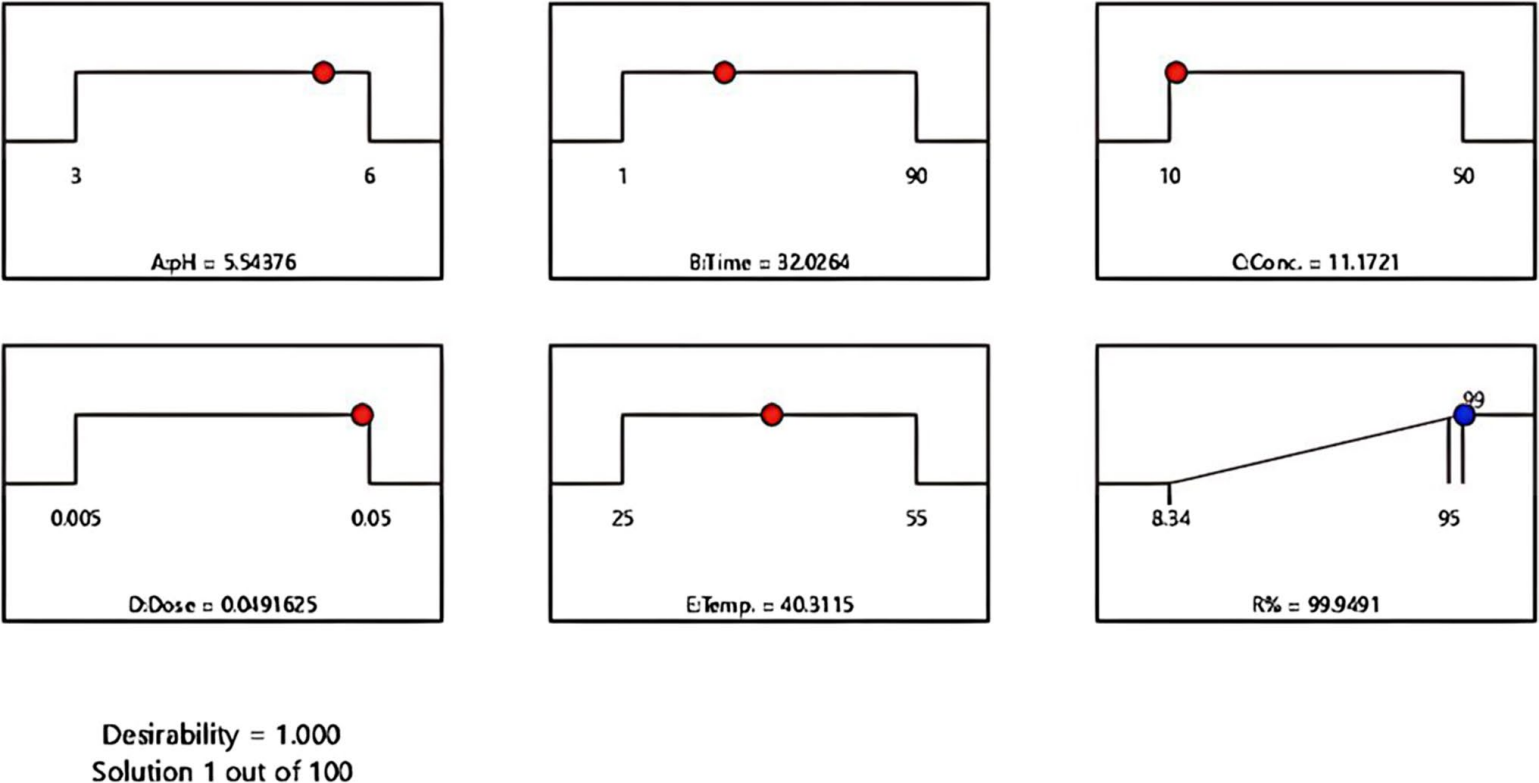
Predicting points of actual profiles regarding the best settings for copper removal

In this investigation, there was just one response taken into account, and the objective was to achieve the highest possible *R*%. The input parameters were each given values that were within a predetermined range (Fig. 6), while the response (*R*%) was considered to achieve a maximum. The ramp desirability is shown in Fig. 6, which was generated using a total of 112 starting points using numerical optimization using the Design Expert software. As observed, the experimental conditions of pH = 5.5, time = 32.0 min, Cu(II) conc. = 11.2 mg L^−1^, dose = 0.05 g, and temp. = 40.3 °C would achieve a *R*% of 99.95 and desirability of 1.000.

### Adsorption studies

#### Effect of the solvent type

To investigate how the kind of solvent affects the prepared adsorbents’ ability to adsorb, MnO_2_ nanostructures were fabricated using different solvents, including isopropyl alcohol, glycerol, and ethylene glycol, where they were denoted as IPA, EG, and GL, respectively. These materials were independently used to prepare MnO_2_@EDTA structures and were labelled IPA-EDTA, EG-EDTA, and GL-EDTA, respectively. These prepared materials were employed to capture Cu(II) ions, and the results are illustrated in Fig. S1. Obviously, there is a slight difference in their removal efficiencies towards Cu(II) ions, where MnO_2_ and MnO_2_@EDTA prepared via isopropyl alcohol exhibited the highest removal percentage. Therefore, MnO_2_@EDTA-based isopropyl alcohol was used to prepare MnO_2_@EDTA-Ag, and both were employed in the succeeding investigations.

#### Effect of initial pH

**Fig. 7:**
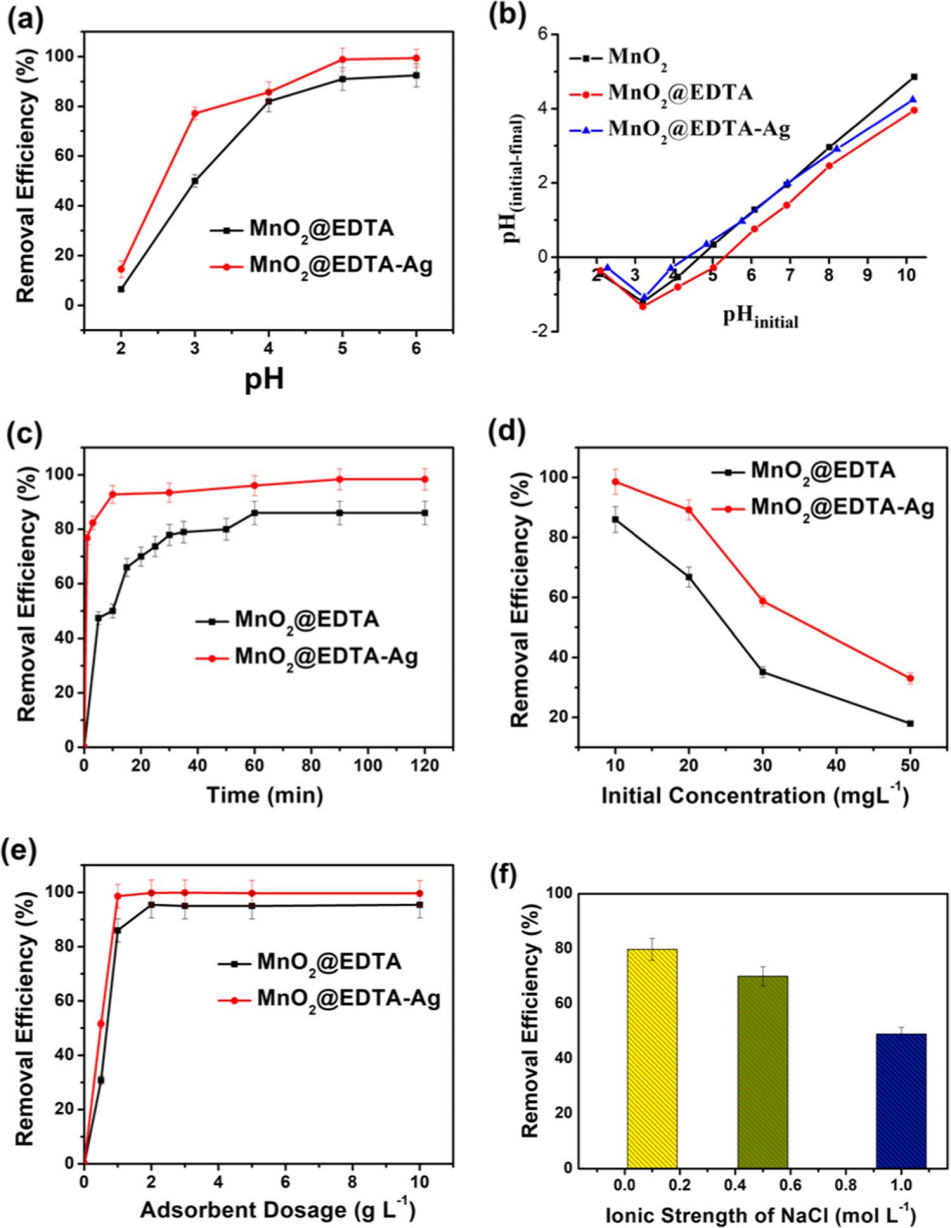
Effect of **a** initial pH, **b** point of zero charge (pH_PZC_), **c** time, **d** initial concentration, **e** adsorbent dosage, **f** ionic strength on Cu(II) removal onto MnO_2_@EDTA and MnO_2_@EDTA-Ag (except **f**, MnO_2_@EDTA-Ag only)). ([Cu^2+^] = 10 mg L^−1^ (expect **b**, **d**), [adsorbent dosage] = 1 g L^−1^ (except **e**), pH = 6 (except **a**, **b**), time = 60 min (expect **c**), and temperature = 25 °C)

Figure 7a illustrates the effect of pH on the Cu(II) adsorption onto the MnO_2_@EDTA and MnO_2_@EDTA-Ag nanocoral reef at initial pH values that varied from 2 to 6. Furthermore, the pH_PZC_ values for MnO_2_, MnO_2_@EDTA, and MnO_2_@EDTA-Ag nanocoral reef (Fig. 7b) were calculated, and they were discovered to be 4.7, 5.3, and 4.3, respectively. It can be noted that as the pH of the Cu(II) solution was raised from 2 to 6, the removal efficiency of the two adsorbents gradually increased. The trend can be interpreted in terms of the electronegativity property of the adsorbents’ surfaces. At low pH values, extra protons and Cu(II) ions competed for the binding sites of the MnO_2_@EDTA and MnO_2_@EDTA-Ag nanocoral reef, resulting in low removal efficiency. As pH values increased, the functional groups on the adsorbents were deprotonated, thereby rendering them more accessible to adsorb Cu(II) ions through electrostatic attraction forces. Based on these observations and the pH_PZC_ values, it can be presumed that electrostatic attraction forces were not the only mechanism for Cu(II) adsorption on the prepared adsorbents. It is possible that several sorption processes, including surface complexation, ion exchange, and precipitation, may be involved in addition to the electrostatic attraction process. These processes have been reported before (Touihri et al. 2021). Hence, pH 6 was utilized in the experiments that followed.

#### Effect of contact time and initial concentration

Figure 7 c shows the removal efficiency (*R*%) of Cu(II) of MnO_2_@EDTA and MnO_2_@EDTA-Ag nanocoral reef as a function of time and constant pH 6. Initially, the Cu(II) adsorption onto MnO_2_@EDTA and MnO_2_@EDTA-Ag was fast. In the first 5 min, 47.4% and 82.3% of Cu(II) were adsorbed onto MnO_2_@EDTA and MnO_2_@EDTA-Ag, respectively. Further, the uptake % of Cu(II) increased gradually up to 120 min with the increasing extension of contact time between adsorbent and adsorbate and afterward sustained a plateau with 86% and 98% removal using MnO_2_@EDTA and MnO_2_@EDTA-Ag, respectively. The rapid initial adsorption rate is responsible for the considerable number of accessible active sites in terms of functional groups and pores on the surface of nanocoral reef in the early stages of the adsorption process. The Cu(II) molecules build up on the surface of the adsorbent as the process of adsorption progresses, blocking molecules from diffusing into the pores and causing a slow adsorption rate (Choudhary et al. 2020). In addition, an improved adsorption behavior from 86 to 98% was achieved using MnO_2_@EDTA and MnO_2_@EDTA-Ag, respectively. Moreover, the initial Cu(II) concentration, which varied between 10 and 50 mg L^−1^ at pH 6.00, had a substantial impact on the adsorption behavior. As shown in Fig. 7 d, when the Cu(II) concentration rose from 10 to 50 mg L^−1^, the Cu(II) removal efficiency (%) onto MnO_2_@EDTA and MnO_2_@EDTA-Ag decreased from 86 and 98% to 18% and 33%, respectively. Insufficient active sites on the adsorbent or a lack of equilibrium time for higher concentration adsorption can be ascribed to the decline in the percentage of Cu(II) removal with increasing initial concentration.

#### Effect of adsorbent dosage

The removal performance (%) of Cu(II) was further investigated using different dosages of adsorbent in the range of 0.5–10 g L^−1^. The Cu(II) concentration was initially 10 mg L^−1^ and was adjusted for an optimal pH of 6.00 in 90 min. As shown in Fig. 7 e, increasing the dosage of adsorbent from 0.5 to 10 g L^−1^ enhanced the removal efficiency (%) of Cu(II) from 30.86 to 95.40% and from 51.9 to 99.8% using MnO_2_@EDTA and MnO_2_@EDTA-Ag, respectively. The elimination (%) of Cu(II) remained essentially constant as the adsorbent dosage was increased to 10 g L^−1^. It was evident that the adsorption efficiency considerably increased with elevating the amount of adsorbent due to the higher number of active sites and porous surfaces (Zare et al. 2015).

#### Effect of ionic strength and interfering ions

Figure 7f presents the effect of ionic strength on the removal (%) of Cu(II) from an aqueous solution. The removal rate (%) dramatically decreased as the NaCl concentration changed, demonstrating the considerable influence of ionic strength on Cu(II) uptake. The effectiveness of Cu(II) adsorption decreased from 98 to 49% when NaCl concentration increased from 0 to 1.00 mol L^−1^. This finding can be accounted for by the development of electrostatic screening between the Cu(II) cations and the negative surface of the MnO_2_@EDTA-Ag adsorbent (Dinh et al. 2020). Additionally, it was examined how different anions, including Cl^−^, NO_3_^−^, HCO_3_^−^, SO_4_^2−^, and different cations, such as Na^+^, K^+^, Mg^2+^, and Ca^2+^, affected the removal (%) of Cu(II) by MnO_2_@EDTA-Ag. The Cu(II) removal efficiency (%) was not notably affected by the existence of Cl^−^, NO_3_^−^, HCO_3_^−^, and SO_4_^2−^. Contrarily, Na^+^, K^+^, Mg^2+^, and Ca^2+^ significantly hindered Cu(II) adsorption; as a result, the percentage of Cu(II) removed from the sample reduced from 98 to 93, 91, 89, and 85%, respectively. The competition between these cations for adsorption sites on the MnO_2_@EDTA-Ag surface was the cause of the observed inhibitory effect.

### Kinetic studies

The rate at which Cu(II) ions are removed using the prepared adsorbents from aqueous solutions can be investigated using kinetic studies, which also provide valuable data for identifying the adsorption mechanism. In this regard, several models are employed, including pseudo-first-order, pseudo-second-order, and intra-particle diffusion models. The linearized forms of the employed kinetic models (Sun et al. 2021) are given in Table S1.

**Fig. 8:**
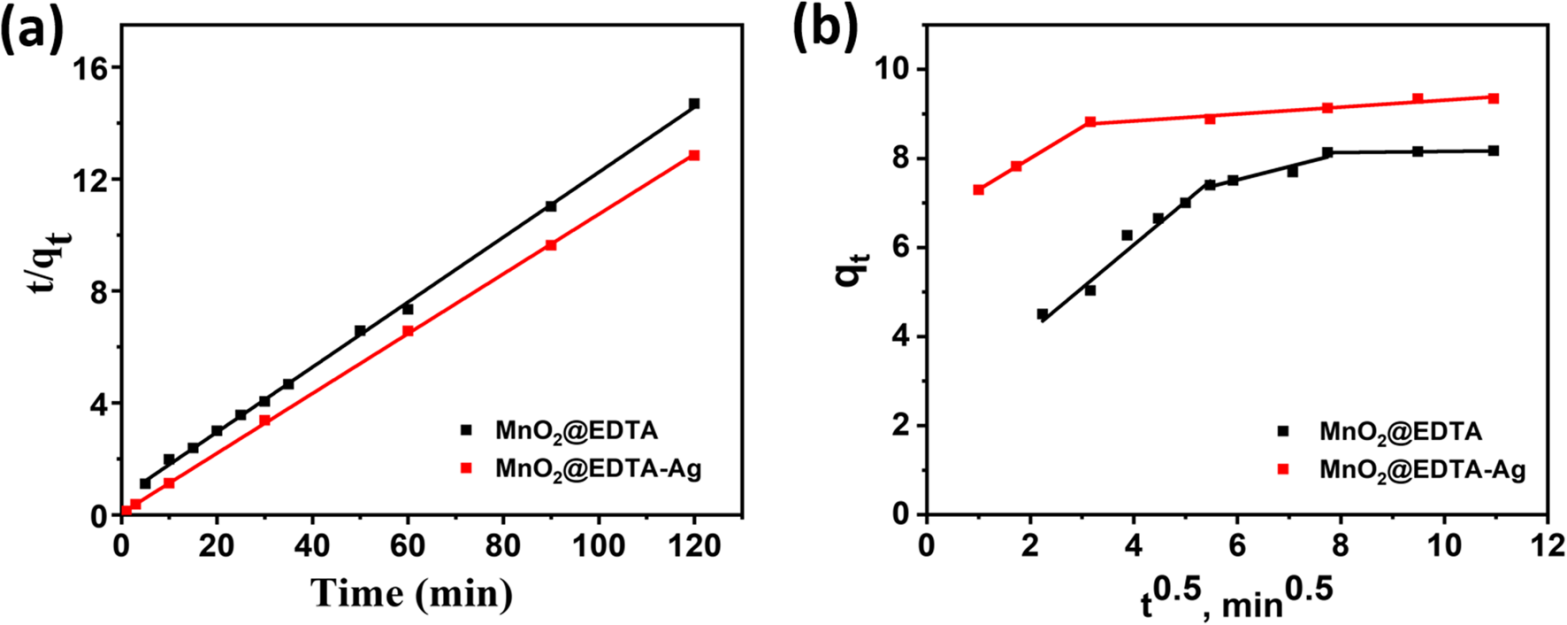
Plots of the pseudo-second-order kinetics (**a**) and intra-particle diffusion (**b**) for Cu(II) removal onto MnO_2_@EDTA and MnO_2_@EDTA-Ag

The kinetic constants and correlation coefficients (*R*^2^) for the applied models were evaluated (Lima et al. 2019), and their corresponding values are demonstrated in Table 4. Also, RMSE, Δ*Q*, and *χ*^2^ (Table S2) were employed to assess the consistency of the computational models and experimental points (Khadhri et al. 2019). As demonstrated by the low values of RMSE, Δ*Q*, and *χ*^2^ and the high values of *R*^2^ (Table 4), pseudo-second-order kinetics (Fig. 8a) rather than pseudo-first-order govern Cu(II) removal onto MnO_2_@EDTA and MnO_2_@EDTA-Ag. Furthermore, the determined *q*_e_ values using the pseudo-second-order model are more consistent with the experimental *q*_e_ values, revealing that the pseudo-second-order kinetics effectively describe the Cu(II) removal using MnO_2_@EDTA and MnO_2_@EDTA-Ag.

**Table 4:** The estimated parameters of the examined kinetic models for Cu(II) removal using MnO_2_@EDTA or MnO_2_@EDTA-Ag

Kinetic model	Parameters	Adsorbents	
		MnO_2_@EDTA	MnO_2_@EDTA-Ag
Pseudo-first-order	*k*_1_	0.074	0.067
	*q*_e (exp)_ (mg g^−1^)	8.17	9.35
	*q*_e (cal)_ (mg g^−1^)	7.16	2.19
	*R*^2^	0.9618	0.9083
	RMSE	0.220	0.399
	Δ*Q*	0.502	0.734
	*χ*^2^	0.410	1.536
Pseudo-second-order	*k*_2_	0.02	0.16
	*q*_e (exp)_ (mg g^−1^)	8.17	9.35
	*q*_e (cal)_ (mg g^−1^)	8.61	9.36
	*R*^2^	0.9991	0.9998
	RMSE	0.121	0.061
	Δ*Q*	0.046	0.118
	*χ*^2^	0.045	0.014
Intra-particle diffusion	*k*_*i*_	0.295	0.078
	*C*	5.75	8.53
	*R*^2^	0.9024	0.9403
	RMSE	0.087	0.055
	Δ*Q*	0.013	0.007
	*χ*^2^	0.004	0.002

Although the pseudo-second-order model provides a sufficiently accurate description of the overall adsorption kinetics, it provides no details on the rate-regulating steps. Therefore, kinetic data were investigated with the Weber and Morris intraparticle diffusion model. To estimate *k*_*i*_ and *C* values, the intercept and slope of the plot of *q*_*t*_ versus *t*^0.5^ were utilized. The plots of this model are shown in Fig. 8b, which reveals the occurrence of two stages in the case of Cu(II) removal using MnO_2_@EDTA-Ag. The initial stage was the film diffusion attributed to the external mass transfer of Cu(II) ions from the bulk solution to the MnO_2_@EDTA-Ag’s surface, while the second stage involved the Cu(II) ions’ passage through the pores of adsorbents (Ali et al. 2020). However, Cu(II) removal using MnO_2_@EDTA comprises three stages, the initial was for film diffusion, while the second was attributed to Cu(II) ions diffusion through MnO_2_@EDTA pores, followed by the last stage at which the system has reached equilibrium. The two plots do not cross the origin point, demonstrating that film diffusion in addition to intra-particle diffusion may play a role in the rate-determining stage for controlling Cu(II) removal using MnO_2_@EDTA or MnO_2_@EDTA-Ag.

### Adsorption isotherms

The Cu(II) ions’ equilibrium concentrations in the solid and liquid phases were expressed using a variety of models, including the Freundlich, Langmuir, Dubinin–Radushkevich (D-R), and Temkin isotherm models (Mozaffari et al. 2020a; Azzam et al. 2022) These models can be expressed in linearized forms, as demonstrated in Table S3.

The isotherms’ constants of the Langmuir and Freundlich models were estimated from their linear plots by plotting *C*_e_/*q*_e_ vs. *C*_e_ and log *q*_e_ vs. log *C*_e_, respectively. Also, plotting ln *q*_e_ vs. ${\upvarepsilon }^{2}$ and *q*_e_ versus ln *C*_e_ enables one to determine the constants *K*_DR_, *B*, and *E* for the D-R model and the constant *b*_T_ for the Temkin model, respectively. Fig. S2 shows the fitting of the experimental data using the applied isotherm models. The calculated parameters of the applied models, along with their *R*^2^, RMSE, Δ*Q*, and *χ*^2^ values, are summarized in Table 5.

**Table 5:** The estimated parameters of the examined isotherm models for Cu(II) removal

Isotherm model	Parameters	Adsorbents	
		MnO_2_@EDTA	MnO_2_@EDTA-Ag
Freundlich	*K*_f_ (mg g^−1^)	7.90	11.16
	*n*	28.64	13.63
	*R*^2^	0.8883	0.9899
	RMSE	0.061	0.063
	Δ*Q*	0.260	1.047
	*χ*^2^	0.023	0.021
Langmuir	*K*_L_ (L mg^−1^)	1.32	2.00
	*q*_max_ (mg g^−1^)	9.24	14.44
	*R*^2^	0.9991	0.9992
	*R*_L_	0.015	0.010
	RMSE	0.046	0.026
	Δ*Q*	0.142	0.849
	*χ*^*2*^	0.013	0.018
D-R	*Β*	0.081	0.016
	*K*_DR_	8.94	13.71
	*E* (kJ mol^−1^)	2.480	5.554
	*R*^2^	0.8423	0.9591
	RMSE	0.053	0.170
	Δ*Q*	0.029	0.086
	*χ*^2^	0.005	0.044
Temkin	*b*_T_ (kJ mol^−1^)	8.255	2.863
	*R*^2^	0.8776	0.9942
	RMSE	0.129	0.132
	Δ*Q*	0.017	0.012
	*χ*^2^	0.008	0.005

It can be inferred from the high values of *R*^2^ and the low values of RMSE, Δ*Q*, and *χ*^2^ (Table 5) that the experimental equilibrium results of Cu(II) ions using MnO_2_@EDTA or MnO_2_@EDTA-Ag are better described by the Langmuir model than the Freundlich model. Also, the *R*_L_ values (between 0 and 1) demonstrated favorable adsorption of Cu(II) onto MnO_2_@EDTA or MnO_2_@EDTA-Ag (Mohamed et al. 2023). Based on the Langmuir isotherm, single-layer adsorption of Cu(II) ions takes place on an even surface of MnO_2_@EDTA or MnO_2_@EDTA-Ag with a limited number of sites for adsorption. In contrast, the Freundlich model is employed for investigating the interaction of adsorbed molecules on heterogeneous surfaces.

The maximum adsorption capacity (*q*_max_) of MnO_2_@EDTA-Ag for copper removal was also compared to those of previous studies, as illustrated in Table 6. As concluded, MnO_2_@EDTA-Ag can be regarded as an effective adsorbent based on comparison with other adsorbents.

**Table 6:** Comparison of the adsorption capacity of MnO_2_@EDTA-Ag nanocoral reef with other adsorbents

Adsorbent	Cu(II) uptake (mg g^−1^)	References
Spent-grain	10.47	Lu and Gibb (2008)
Powdered banana peel waste	3.29	Seleman et al. (2023)
Chitosan/ZnO nanorod composite	8.01	Ali and Mohamed (2017)
Rubber leaves powder	9.07	Rukayat et al. (2021)
Alg/MWCNTs/DAC nanocomposite	26.6	Eldeeb et al. (2021)
PMMA/SilicaKit6	9.03	Dinari et al. (2016)
Coated chitosan on alumina	86.2	Boddu et al. (2008)
Ni ferrite-modified montmorillonite	18.73	Ahmed et al. (2021)
Carbon gel doped with graphite	8.64	Osińska (2017)
CS-SiO_2_@TEuTTA membrane	51.28	Li et al. (2023)
PBAT microplastics	0.192	Sun et al. (2023)
ALG-HT biocomposite	63.25	Menye et al. (2023)
CuO-modified ceramic membrane	13.03	Mahatmanti et al. (2023)
MnO_2_@EDTA-Ag	14.44	This work

To identify the kind of adsorption—physisorption or chemisorption—the equilibrium data were modeled applying the D-R isotherm. As noticed from Table 5, Cu(II) adsorption onto MnO_2_@EDTA and MnO_2_@EDTA-Ag was physical in nature because the obtained values of *E* were < 8 kJ mol^−1^ (Kakavandi et al. 2015). Moreover, the Temkin constant (*b*_T_) has been determined to provide further details on the physical nature of Cu(II) adsorption onto the prepared adsorbents. It was found that the values of *b*_T_ were 8.255 and 2.863 kJ mol^−1^ for MnO_2_@EDTA and MnO_2_@EDTA-Ag, respectively, revealing weak electrostatic interactions (Mohammadnezhad et al. 2017).

### Thermodynamic studies

At various temperatures (298–328 K), the sorption process’ thermodynamic characteristics were assessed. The following equations were used to estimate the enthalpy change (∆*H*, kJ mol^−1^), entropy change (∆*S*, J mol^−1^ K^−1^), and free energy change (∆*G*, kJ mol^−1^) (Ali and Mohamed 2017):

5 $${K}_{\mathrm{d}} = \frac{{q}_{\mathrm{e}}}{{C}_{\mathrm{e}}}$$

6 $$\Delta G=-\mathrm{RT}\;\mathrm{In}\;K_d$$

7 $$\ln\;K_{\mathrm d}=\frac{\Delta S}R-\frac{\Delta H}{\mathrm{RT}}$$

where *R* is the universal gas constant (8.314 J K^−1^ mol^−1^), T is the temperature in Kelvin, and *K*_d_ is the equilibrium constant. The slope and intercept of the plot of ln *K*_d_ vs 1/T can be used to determine the values of ∆*H* and ∆*S*.

As shown in Table 7, for the adsorption of Cu(II) onto MnO_2_@EDTA and MnO_2_@EDTA-Ag, the positive and negative values of ∆*H* revealed that the nature of the adsorption process was endothermic and exothermic, respectively. When the value of ∆*H* is less than 80 kJ mol^−1^, the adsorption process involves physisorption, such as van der Waals or electrostatic interaction, and when the value of ∆*H* is greater than 80 kJ mol^−1^, the interaction is chemisorption (Ali and Mohamed 2017). The adsorption was favorable since the positive values for ∆*S* suggested a rise in randomness at the solid-solution interface. The disorder at the solid/liquid boundary (interface) was reduced throughout the adsorption process of the copper ions, as shown by the negative value of ∆*S* (− 128.82 J K^−1^ mol^−1^) obtained using MnO_2_@EDTA-Ag. Because copper ions are more mobile at higher temperatures, ions from the solid phase were able to move into the liquid phase. Therefore, less amount of copper could be adsorbed (Rukayat and Usman 2021). The negative Δ*G* values at the higher temperatures demonstrated the viability and spontaneity of the Cu(II) sorption process onto MnO_2_@EDTA, whereas the positive Δ*G* values at the lower temperatures reflected the non-spontaneous character of the adsorption process. On the contrary, Cu(II) adsorption onto MnO_2_@EDTA-Ag was spontaneous at the lower temperatures and non-spontaneous at the higher ones.

**Table 7:** Thermodynamic parameters for Cu(II) removal using MnO_2_@EDTA and MnO_2_@EDTA-Ag adsorbents

Adsorbent	△*H* (kJ mol^−1^)	△*S* (J mol^−1^ K^−1^)	△*G* (kJ mol^−1^)			
			298 K	308 K	318 K	328 K
MnO_2_@EDTA	33.56	107.25	1.482	0.409	− 0.662	− 1.735
MnO_2_@EDTA-Ag	− 38.73	− 128.82	− 0.349	0.938	2.226	3.514

### Desorption and reusability study

**Fig. 9:**
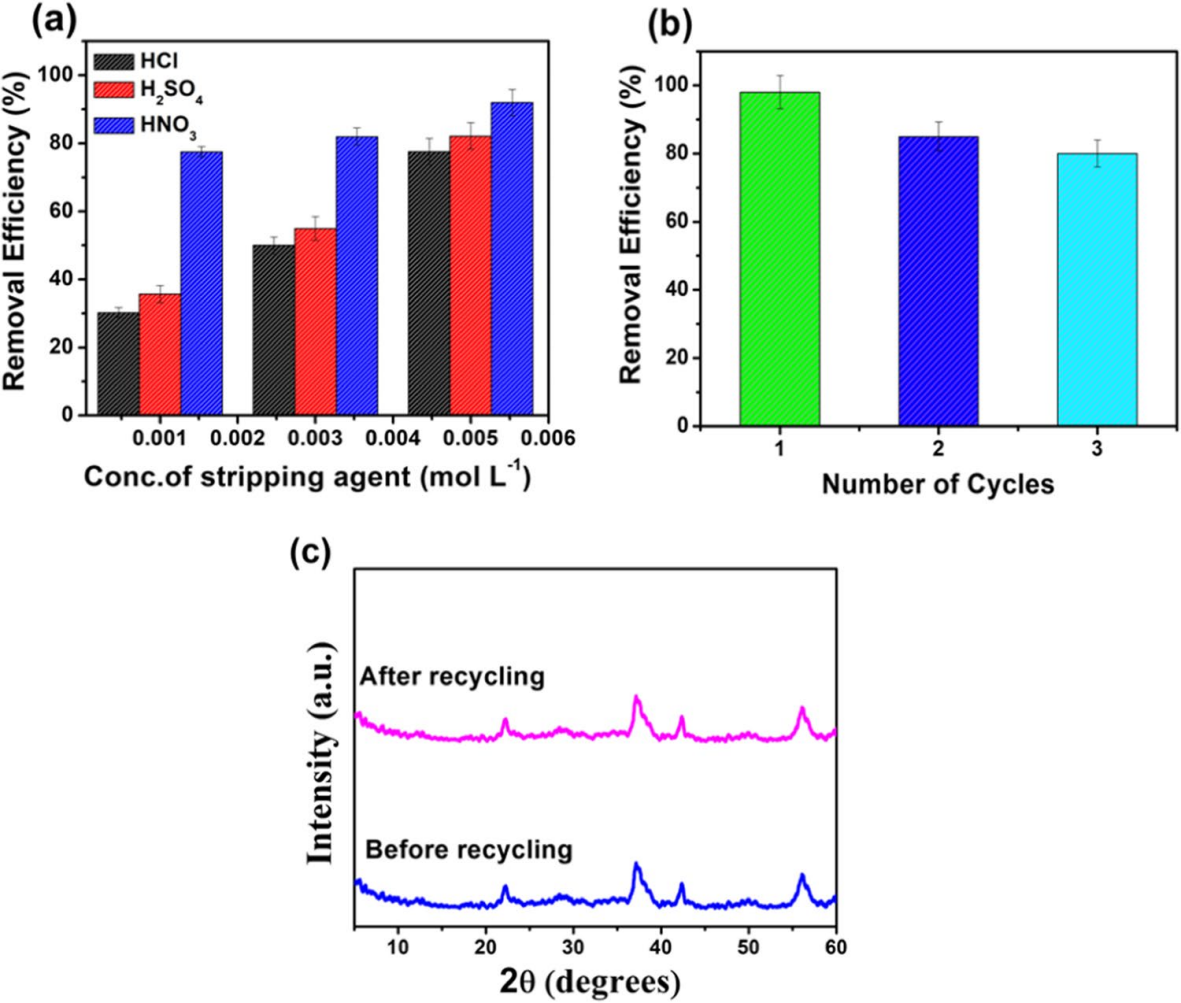
**a** Effect of stripping agents for desorption study, **b** recycling of MnO_2_@EDTA-Ag for Cu(II) removal, and **c** XRD patterns of MnO_2_@EDTA-Ag before and after the third cycle

To evaluate the desorption behavior of MnO_2_@EDTA-Ag, different concentrations of strong acids, such as HCl, H_2_SO_4_, and HNO_3_, ranging from 0.001 to 0.005 mol L^−1^ are most commonly used to elute Cu(II). It was observed that increased concentration of strong acids (0.001–0.005 mol L^−1^) enhanced the desorption efficiency (%) of Cu(II) from 30.24 to 77.55%, 35.66 to 82.16%, and 77.50 to 92% using HCl, H_2_SO_4_, and HNO_3_, respectively (Fig. 9a). For economic reasons and environmental points of view, recycling of adsorbents has a significant role. Herein, 0.005 mol L^−1^ HNO_3_ was used as a stripping agent for regeneration of MnO_2_@EDTA-Ag. After the adsorption process of Cu(II), MnO_2_@EDTA-Ag was recollected and mixed with 10 mL HNO_3_ (0.005 mol L^−1^), shaken for 1 h, and separated. The collected adsorbent was washed with distilled water and dried to be reused again. The Cu(II) solution was added again to the collected adsorbent to evaluate the stability of the adsorbent. After completing three consecutive cycles of adsorption–desorption, the efficacy of MnO_2_@EDTA-Ag for removing Cu(II) was slightly reduced, as shown in Fig. 9b. The removal efficiency % of the Cu(II) solution was reduced from 98 to 80%. In addition, there is no significant change in the XRD analysis before and after the recycling process, implying its stability (Fig. 9c). The results demonstrated that the MnO_2_@EDTA-Ag nanocoral reef was comparably stable under the studied conditions, which is an advantage of using the adsorbent for environmental remediation and the industrial field.

### Proposed removal mechanism of Cu(II) onto MnO_2_@EDTA-Ag

**Fig. 10:**
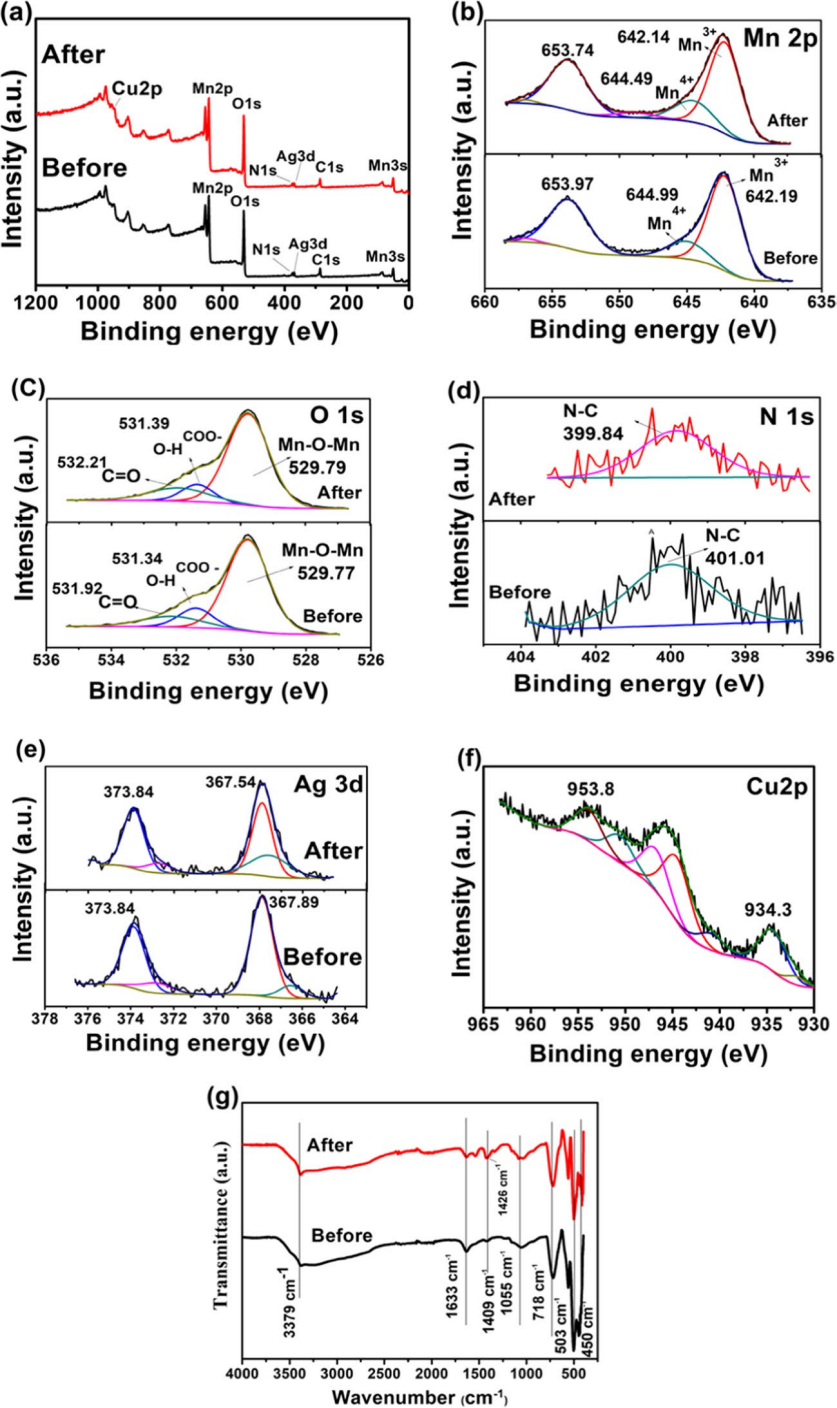
XPS and FTIR analysis before and after adsorption of Cu(II) onto MnO_2_@EDTA-Ag: **a** full survey, **b** Mn 2p, **c** O 1s, **d** N 1s, **e** Ag 3d, **f** Cu 2p, and **g** FTIR spectra

The adsorption mechanism has been comprehensively elucidated using XPS and FTIR analysis. The chemical states of MnO_2_@EDTA-Ag nanocoral reef before and after Cu(II) adsorption were investigated by XPS analysis. Figure 10a illustrates the XPS survey spectra of both samples before and after Cu(II) removal, where the typical Mn, Ag, C, O, and N signals could be observed. Meanwhile, the newly appeared peak of the Cu(II) signal could be found in the sample after adsorption, confirming the uptake of Cu(II) ions onto the MnO_2_@EDTA-Ag nanocoral reef. The high-resolution scan of the Mn 2p spectrum is given in Fig. 10b. The peaks located around binding energies of 642.14 and 653.74 eV are assigned to Mn 2p_3/2_ and Mn 2p_1/2_. The Mn 2p_3/2_ and Mn 2p_1/2_ peaks showed a splitting of 11.55 eV, which confirms that the main oxidation state of Mn atoms is+4. The values of binding energy at 642.19 and 644.99 eV of the Mn 2p_3/2_ spectrum are ascribed to Mn^3+^ and Mn^4+^, respectively. After Cu(II) adsorption, the binding energy of the Mn 2p_3/2_ spectrum is slightly shifted to 642.14 and 644.49 eV, confirming the absence of Mn^2+^ in the MnO_2_@EDTA-Ag nanocoral reef. In addition, the Mn^3+^/Mn^4+^ ratio of MnO_2_@EDTA-Ag nanocoral reef is slightly increased from 3.40 to 4.38% after the adsorption process, confirming the absence of redox reactions (Lin and Chen 2020). As shown in Fig. 10c, the O 1 s spectrum before and after adsorption was elucidated. The peak located at 529.77 eV is attributed to lattice oxygen of metal oxide (Mn–O–Mn bonds), the peak at 531.34 eV corresponds to Mn–OH and –COO^−^ groups, and the peak at higher binding energy, 531.92 eV, associated with OH groups of water molecules or carbonyl groups (Claros et al. 2021). After adsorption of Cu(II) to the surface of MnO_2_@EDTA-Ag, the peaks of 531.34 and 531.92 eV are shifted to 531.39 and 532.12 eV, respectively. The relative area ratio for the peak of the Mn–O bond is slightly increased from 74.21 to 74.24% due to the binding of Mn–O, Cu–O, and the formation of bidentate mononuclear or multidentate complexes, instead of monodentate complexes during the adsorption. Meanwhile, the relative ratio of Mn–OH decreased from 13.06 to 10.58% due to the participation of the hydroxyl groups in the adsorption process (Lin and Chen 2020; Claros et al. 2021). Before the adsorption of Cu(II), the value of binding energy of N 1s spectrum (Fig. 10d) was 401.01 eV, which is slightly shifted to 399.84 eV after the adsorption process, suggesting that Cu(II) ions may be bound to N atoms of EDTA by chelation interaction (Cui et al. 2021). Therefore, the synergistic effect between the physisorption forces and the chelation interaction of MnO_2_@EDTA-Ag nanocoral reef enhanced Cu(II) removal. The Ag 3d spectrum (Fig. 10e) before and after Cu(II) adsorption exhibits only a couple of peaks at binding energies of 367.89 and 373.84 eV, corresponding to Ag 3d_5/2_ and Ag3d_3/2_, respectively, showing the presence of metallic Ag (NPs) (Bai et al. 2016; Tang et al. 2019). The relative area ratio of Ag 3d_5/2_ decreased from 52.91% (before adsorption) to 38.52% (after adsorption). This decrease may be due to the adsorption of Cu(II) onto MnO_2_@EDTA-Ag. The binding energies at 934.3 and 953.8 eV (Fig. 10f) are attributed to Cu 2p_3/2_ and Cu 2p_1/2_, with a spin–orbit separation of 19.5 eV, implying the removal of Cu(II) ions onto MnO_2_@EDTA-Ag nanocoral reef (Claros et al. 2021). In addition, the “shake-up” satellite peaks in the range 940–945 eV are evidence for the presence of Cu^2+^ state (Mulla and Rabinal 2021).

Additionally, Fig. 10g displays the MnO_2_@EDTA-Ag FTIR spectra before and after Cu(II) capture. Following Cu(II) adsorption, the peak at 1409 cm^−1^ ascribed to the symmetric stretching vibration of C–N bonds and COO^−^ groups that originated from EDTA shifted to 1426 cm^−1^. This implies that these groups participate in the formation of a coordination complex involving Cu(II).

### Environmental applications

The analytical usefulness of MnO_2_@EDTA-Ag to be employed for water remediation was demonstrated by applying them to remove Cu(II) ions that were spiked in different water samples, including groundwater, Nile water, and tap water. This was carried out by shaking 0.01 g of MnO_2_@EDTA-Ag for 1 h, at pH 6 with 10 mL of each water sample that was previously spiked with 10 mg mL^−1^ Cu(II). The results are presented in Fig. S3, which revealed no significant difference in the removal efficiency of MnO_2_@EDTA-Ag in any of the water samples tested, reflecting the effectiveness of the prepared samples in wastewater treatment. It is worth mentioning that the equilibrium Cu(II) concentration in all the tested samples was all less than 0.7 mg L^−1^, demonstrating the remarkable effectiveness of MnO_2_@EDTA-Ag to remediate complex wastewater from heavy metals.

## Conclusion

In this work, a novel MnO_2_@EDTA-Ag nanocoral reef was effectively synthesized via a facile redox reaction followed by impregnation with EDTA and silver nanoparticles for rapid removal of hazardous copper (II) from real water samples. The results showed that the nanocoral reef structure’s Cu(II) removal efficiency (99.95%) employing Plackett–Burman design (PBD) was achieved at pH 5.5, a contact time of 32.0 min, a Cu(II) concentration of 11.2 mg L^−1^, MnO_2_@EDTA-Ag dose of 0.05 g, and a temperature of 40.3 °C. The proposed model with PBD revealed that the dose of MnO_2_@EDTA-Ag, followed by Cu(II) concentration and pH, were the most significant parameters affecting Cu(II) removal. It was found that the loading of Ag NPs onto MnO_2_@EDTA improved the adsorption capability of MnO_2_@EDTA-Ag. The adsorption data were significantly correlated with the models of pseudo-second-order kinetics and Langmuir isotherm. Moreover, the thermodynamic study of the Cu(II) removal is energetically exothermic. XPS and FTIR analysis elucidated that the electrostatic interaction and chelation/complexation are responsible for the removal mechanisms of Cu(II). No significant loss in the Cu(II) removal efficiency using the real wastewater samples compared to the aqueous solution was observed, revealing its efficacy in water remediation. Additionally, the recyclability of MnO_2_@EDTA-Ag nanocoral reef for Cu(II) was maintained at 80% after three adsorption–desorption cycles, and the XRD data before and after the recycling process revealed no significant changes, suggesting its stability. According to these conclusions, the MnO_2_@EDTA-Ag nanocoral reef shows promise as a sorbent for the removal of potentially hazardous metals from wastewater.

### Supplementary Information

Below is the link to the electronic supplementary material.

Supplementary file1 (DOCX 1.62 MB)

## Data Availability

Raw data is available upon request.
