# Supplementary material for: Functional Ag-EDTA-modified MnO2 nanocoral reef for rapid removal of hazardous copper from wastewater
Source: Environ Sci Pollut Res Int. 2023 Nov 22;30(59):123751–69. doi: 10.1007/s11356-023-30805-0 (PMC10746771; doi:10.1007/s11356-023-30805-0)
Supplement: Supplementary file 1 — Supplementary file1 (DOCX 1.62 MB) [file 11356_2023_30805_MOESM1_ESM.docx]

**Supporting information**

# Functional Ag-EDTA-modified MnO_2_ nanocoral reef for rapid removal of hazardous copper from wastewater

Omnia I. Ali ^a ,^* and Ahmed B. Azzam ^a^

^a^Chemistry Department, Faculty of Science, Helwan University, 11795, Cairo, Egypt

* Corresponding author: Tel.: 002 01002583552; fax: 00202 25552468

E-mail addresses: [omniaali@science.helwan.edu.eg](mailto:omniaali@science.helwan.edu.eg) & [omniaibrahim95@gmail.com](mailto:omniaibrahim95@gmail.com)

ORCID/ 0000-0002-4205-0060

# 2. Experimental

## *2.1. Materials*

Manganese sulfate hydrate (MnSO_4_.H_2_O), potassium permanganate (KMnO_4_), silver nitrate (AgNO_3_), isopropyl alcohol, glycerol, and ethylene glycol were purchased from Sigma-Aldrich and used in this work without further purification. Sodium diethyldithiocarbamate AR was purchased from Loba-Chemie, India. A 1000 mg L^-1^ of Cu(II) stock solution was prepared by dissolving 3.928 g of CuSO_4_.5H_2_O (Merck) in 1.0 L double distilled water. All stock solutions are prepared with double distilled water.

## *2.2. Characterization*

X-ray powder diffraction (XRD) patterns were recorded using a Brucker, Axs D8-advance diffractometer with cupper inner shell-Kα radiation (λ= 1.5406 Å) at 2θ ranging from 5° to 60° and scan rate of 2° min^-1^. Quanta FEG-250 SEM with 20 kV accelerated voltage and Transmission electron microscopy (TEM, JEM-2100, Japan) with 200 kV are used to illustrate the surface morphologies of as-prepared materials. The valence state and elemental composition of modified MnO_2_ were characterized using Thermo Scientific™ K-Alpha™ XPS spectrometer, Al-Kα Micro focused monochromator within an energy range up to 4 KeV. The Fourier transform infrared (FTIR) spectra were recorded by PerkinElmer (model spectrum one FT-IR spectrometer, USA) spectrometer (400–4,000 cm^-1^). The contents of copper ions in the solution were analyzed by UV-Vis Spectrophotometer, Jasco-V-570. A digital pH meter (Jenco 6173) was used to measure the pH of the working solutions.

**
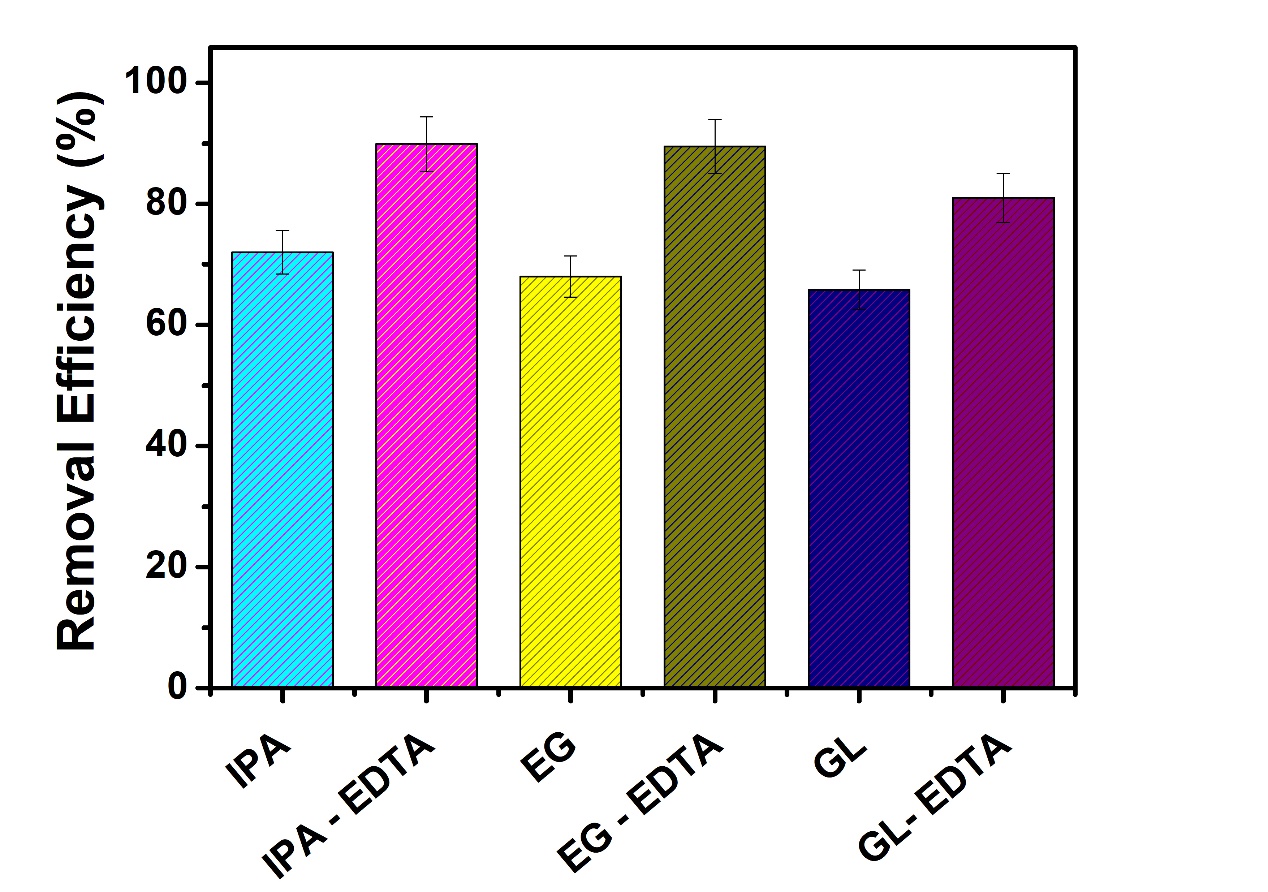
**

**Fig. S1** Effect of solvent type on Cu(II) removal onto MnO_2_ nanostructures samples ([Cu^2+^] = 10 mg L^-1^, [adsorbent dosage] = 1 g L^-1^, pH= 6, time = 60 min, and temperature = 25 °C).

**(a)**

**(b)**

**(c)**

**(d)**

**Fig. S2** Plots of Freundlich (a), Langmuir (b), D-R (c), and Temkin (d) isotherm models for Cu(II) removal onto MnO_2_@EDTA and MnO_2_@EDTA-Ag.

**
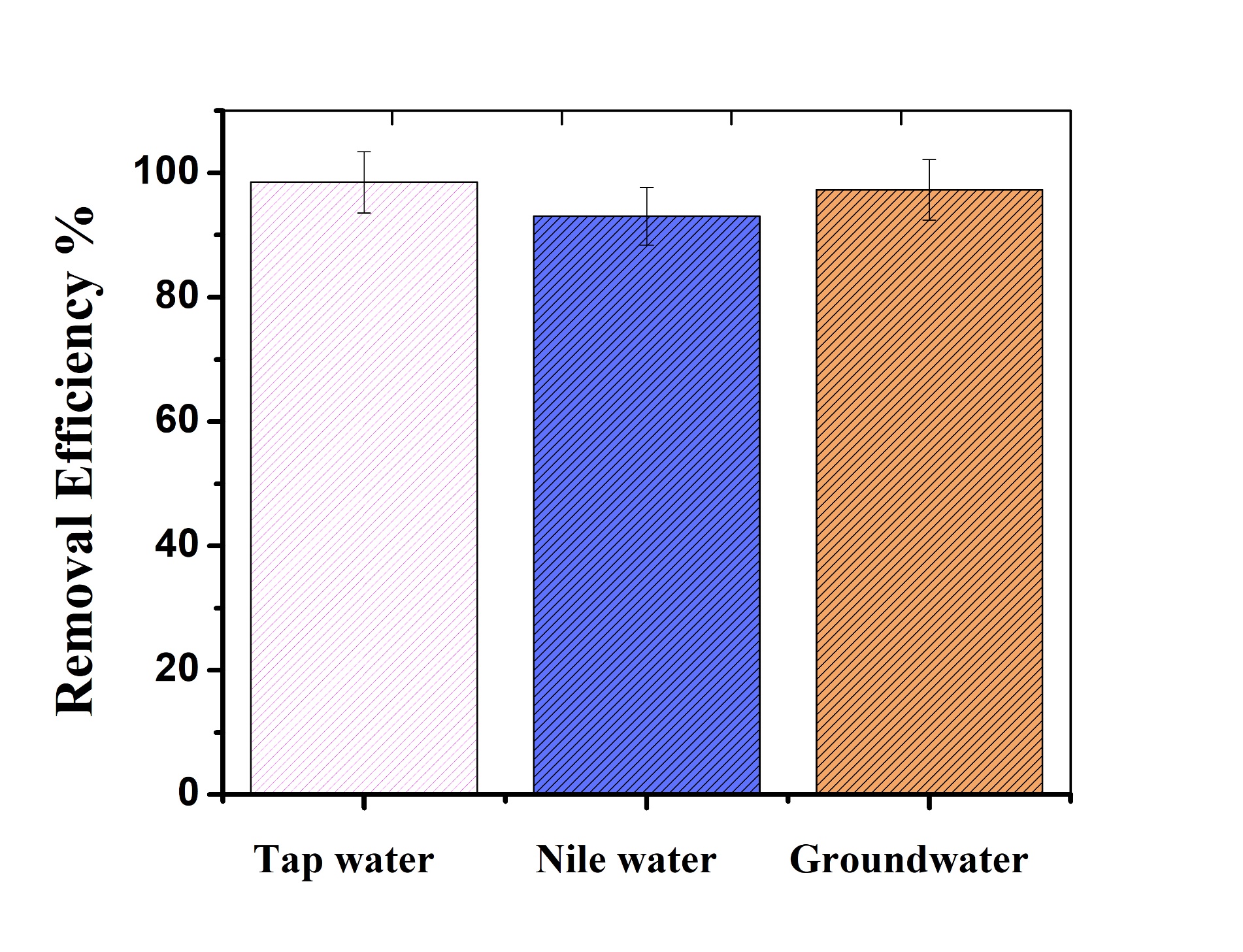
Fig. S3** Cu(II) removal from real water samples using MnO_2_@EDTA-Ag.

**Table S1.** Mathematical forms of applied kinetic models.

| Kinetic Model | Linear Equation |
| --- | --- |
| Pseudo-first-order | log (q_e_ – q_t_) = log q_e_ – $\frac{k_{1}}{2.303}t$ |
| Pseudo-second-order | $\frac{t}{q_{t}}= \frac{1}{k_{2}q_{e}^{2}}+ \frac{t}{q_{e}}$ |
| Intra-particle diffusion | $q_{t}= {k_{i} t^{0.5}}+C$ |

where q_e_ (mg g^-1^) and q_t_ (mg g^-1^) are the adsorption capacities at equilibrium and at time t (min), respectively; k_1_ (min^-1^), k_2_ (g mg^-1^ min^-1^), and k_i_ (mg g^-1^ min^-1/2^) are the rate constants of pseudo-first-order, pseudo-second-order, and the intraparticle diffusion models, respectively; and C provides information about the limit layer's thickness.

**Table S2.** Expressions of error functions.

| Function | Equation* |
| --- | --- |
| Residual root-mean square error | RSME = $\sqrt{\frac{\sum_{i=1}^{n} \left( q_{exp}-q_{mod} \right)^{2}}{n}}$ |
| Normalized standard deviation | ΔQ = $\sqrt{\frac{\sum_{i=1}^{n} \left( \left( q_{exp}-q_{mod} \right)/q_{exp} \right)^{2}}{n-1}}$ |
| Chi-squared | χ^2^ = $\sum\left( \frac{\left( q_{exp}-q_{mod} \right)^{2}}{q_{mod}} \right)$ |
| * *q_exp_* (mg g^-1^) is the experimental value, *q_mod_* (mg g^-1^) is the value as calculated from the model, n is the number of experimental data points. | |

**Table S3.** Mathematical forms of applied equilibrium isotherm models.

| Isotherm Model | Linear Equation |
| --- | --- |
| Langmuir | $\frac{C_{e}}{q_{e}}= \frac{C_{e}}{q_{\max}}$ + $\frac{1}{q_{\max} K_{L}}$ |
| Freundlich | log q_e_ = log K_F_ +$\frac{1}{n}$log C_e_ |
| Dubinin–Radushkevich (D-R) | ln $q_{e}$ = B$\varepsilon^{2}$ + ln $K_{\mathrm{DR}}$  ε = RT ln [ 1 + $\frac{1}{C_{e}}$ ]  E = (2B)^-0.5^ |
| Temkin | $q_{e}=\frac{\mathrm{RT}}{b_{T}}\ln K_{T} + \frac{\mathrm{RT}}{b_{T}}\ln C_{e}$ |

where q_max_ is the maximum adsorption capacity (mg g^-1^), K_L_ is the Langmuir constant, K_F_ and n are constants signifying the uptake capacity (mg g^−1^) and intensity (unitless), respectively, the D-R optimum monolayer adsorption capacity is symbolized by K_DR_ (mg g^-1^), ε is the Polanyi potential, the activity coefficient with respect to the mean adsorption energy is B (mol^2^ kJ^-2^), E (kJ mol^-1^) is the mean sorption free energy, K_T_ is the Temkin constant (L g^-1^) correlating to the maximum binding energy, b_T_ is related to the sorption heat (J mol^-1^), R is the universal gas constant (8.314 J mol^-1^ K^-1^), and T is the temperature (K).
